# HER2 Positivity Is Affected by the Papillary Structure and Has a Bidirectional Prognostic Value for Gallbladder Carcinoma

**DOI:** 10.3389/fgene.2021.831318

**Published:** 2022-02-04

**Authors:** Lingli Chen, Lei Xu, Licheng Shen, Rongkui Luo, Dongxian Jiang, Yueqi Wang, Wei Li, Yingyong Hou

**Affiliations:** ^1^ Department of Pathology, Zhongshan Hospital, Fudan University, Shanghai, China; ^2^ Department of General Surgery, Zhongshan Hospital, Fudan University, Shanghai, China; ^3^ Department of Oncology, Zhongshan Hospital, Fudan University, Shanghai, China

**Keywords:** gallbladder carcinomas, papillary adenocarcinoma, prognosis, HER2, HER2 positivity

## Abstract

Gallbladder carcinoma (GBC) is responsible for 80%–95% of biliary tract malignancies and has a dismal prognosis. Human epidermal growth factor receptor 2 (HER2) is a promising therapeutic target of GBC. Through immunohistochemistry (IHC) and fluorescence *in situ* hybridization (FISH) methods, HER2 expression and gene amplification were identified on high-output tissue microarrays (TMAs) developed in 306 GBC cases to investigate its relationship with GBC and clinicopathological characteristics. Adenocarcinomas accounted for 223 (72.9%) of the cases, with 62 (27.8%) being papillary adenocarcinoma or having partial papillary structure. HER2 positivity was studied in 16.1% (36/223) of patients with adenocarcinoma and 41.9% (26/62) in adenocarcinoma with papillary structures. For 143 radically resected primary GBC cases with 24 HER2-positive tumors, survival data were valid; the median survival time was not reached, and the 5-year survival rate was 52.9%. All patients in stages 0–I survived, and the results of the HER2-positive group and the stage II HER2-negative group were similar (*p* = 0.354). However, in stage III, the mortality rate in the HER2-positive group was reduced (*p* = 0.005) and that in stage IV was higher (*p* = 0.005). In conclusion, HER2 positivity was significantly higher in patients with papillary GBC. The predictive value of HER2 varies by clinical stage, with no prediction in the early stages, better in stage III, and worse in stage IV.

## Background

Human epidermal growth factor receptor 2 (HER2), alias Neu or ErbB2, is an important oncogene that has an essential function in cell proliferation and dedifferentiation (Yarden and Sliwkowski, 2001) and has also been extensively studied in breast (Slamon et al., 2001; Slamon et al., 2011), gastric (Bang et al., 2010), and colon cancer (El-Deiry et al., 2015). HER2 positivity is responsible for probably 15%–20% of breast cancer and remains the only predictive factor for the selection of targeted therapies, except for hormone receptors (Loibl and Gianni, 2017). Overexpression or amplification of HER2 is thought to be present in 15%–20% of advanced gastric and gastroesophageal junction cancers (Van Cutsem et al., 2015). Chemotherapy plus the anti-HER2 antibody trastuzumab is the recommended first-line therapy (Muro et al., 2019) based on the randomized, multicenter, phase 3 ToGA (Trastuzumab for Gastric Cancer) trial, in which the overall survival (OS) was significantly longer with chemotherapy plus trastuzumab than with chemotherapy alone (Bang et al., 2010).

Gallbladder carcinoma (GBC) represents 80%–95% of patients with biliary tract malignancy and ranks fifth in digestive cancer. Since most cases are in the advanced stage, GBC often leads to a poor prognosis and a less than 1-year median survival rate (Lazcano-Ponce et al., 2001). Even with the current first-line standard-of-care treatment (gemcitabine–cisplatin) for advanced GBCs, the median OS is less than 1 year (Valle et al., 2014). Thus, to improve the prognosis in GBC, a new therapeutic target is required, and HER2 is a promising approach. In the MyPathway basket trial, HER2-positive tumors were treated with pertuzumab and trastuzumab (Hainsworth et al., 2018). Thirty (26%) of 114 patients with HER2 amplification/overexpression had objective responses. The study included seven biliary cancers/GBCs, and the objective response rate was 29% (2/7) (95% CI = 4–71). However, current data for HER2-positive ratio present in GBC are contradictory. HER2 status in GBC shows considerable heterogeneity, and the frequency of the HER2-positive ratio varies from 0% to 31.3% in different studies (Albrecht et al., 2020; Hiraoka et al., 2020).

In the present study, a large, well-characterized cohort of patients with GBC was established. HER2 status was determined in this cohort with the recommended testing guidelines for gastric cancer using a combination of fluorescence *in situ* hybridization (FISH) and immunohistochemistry (IHC) methods. The purpose of this study was to study in depth the HER2 status of GBC patients and assess whether HER2 is related to clinicopathological characteristics and prognosis.

## Materials and Methods

### Sample and Tissue Selection

All study participants provided informed consent before starting the study, and the study design obtained approval from the Ethics Committee of Zhongshan Hospital, Fudan University. Between 2013 and 2018, a total of 306 patients with primary GBC who underwent surgery at Zhongshan Hospital participated in the study. Clinicopathological parameters, such as sex, age, type of surgery, histological classification, TNM classification, and American Joint Committee on Cancer 8th stage, were reviewed. The World Health Organization’s 5th classification was applied for histological categorization. Patient histology slices were reviewed, and blocks with sufficient representative tissue were chosen, avoiding areas of massive necrosis, to construct high-output tissue microarrays (TMAs) (Shi et al., 2013). Serial tissue sections of 4 μm were adopted for hematoxylin and eosin staining, IHC, and FISH.

### IHC and FISH

HER2 IHC was conducted using anti-HER2 antibodies, an automated slide stainer, and iView DAB detection kit (Ventana Medical System, Tucson, AZ, United States). HER2 IHC scoring was based on gastric cancer. As IHC was applied on TMAs, if stained tumor cells were <10%, the whole block would be stained.

In the HER2 IHC scoring criteria (Hofmann et al., 2008), no tumor cell staining or <10% was recorded as 0. Faint membranous staining in more than 10% of tumor cells was recorded as 1+. A score of 2+ indicates weak to moderate complete or basolateral membranous staining in over 10% of tumor cells, while 3+ indicates moderate to strong staining.

FISH was conducted using Pathvysion HER2 DNA Probe Kit (Abbott Laboratories, Abbott Park, IL, United States) on the GBC TMAs following the manufacturer’s instructions.

HER2 positivity represented either a 3+ or a 2+ IHC score with positive FISH results (*HER2* gene amplification).

### Follow-Up

All patients were followed up, but patients with the following conditions were excluded from survival analysis: 1) if they received neoadjuvant chemotherapy and/or radiation; 2) had other accompanying malignant tumors (such as gastric or colon cancer); 3) had non-radical surgery; and 4) perioperative death. A total of 146 patients who received radical surgery and were diagnosed with adenocarcinoma (AC) [otherwise signet cell carcinoma/poorly cohesive carcinoma or mucinous adenocarcinoma (MAC)] were included in the survival analysis. The survival data for 143 patients were obtained.

### Statistical Analysis

The *χ* ^2^ test was used to analyze categorical data. For parametric data, Student’s *t*-test was used for comparing two means. Survival curves were analyzed with the Kaplan–Meier method. During the survival analysis, cases that were not followed up and fatalities that were not caused by GBC were censored. The log-rank or Breslow’s tests was used to assess the significance of discrepancies in the survival curves. SPSS (version 22.0) was utilized for all statistical data processing. A *p* < 0.05 was considered to denote statistically significant differences.

## Results

### Overexpression of the HER2 Protein

The HER2 IHC results for 306 GBCs are shown in Table 1. In total, 18 were classified with a 3+ score (5.9%), 37 were classified with a 2+ score (12.1%), 26 were classified with a 1+ score (8.5%), and 225 (73.5%) cases were classified with a score of 0.

**TABLE 1:** HER2 overexpression and amplification of 306 gallbladder carcinomas

		HER2 expression (IHC)				Total
		0	1	2	3	
*HER2* gene status	Not amplified	222 (84.1%)	23 (8.7%)	19 (7.2%)	0	264
	Amplified	3 (7.1%)	3 (7.1%)	18 (42.9%)	18 (42.9%)	42
Total		225 (73.5%)	26 (8.5%)	37 (12.1%)	18 (5.9%)	306

HER2, human epidermal growth factor receptor 2; IHC, immunohistochemistry

### *HER2* Gene Amplification

The results of *HER2* gene amplification in 306 cases assessed using FISH are shown in Table 1. Overall, 42 (13.7%) patients exhibited *HER2* amplification. All 18 (100%) tumors with a 3+ immunostaining score exhibited *HER2* gene amplification. Among the 37 tumors with a 2+ immunostaining score, 18 (48.6%) showed amplification. Six (2.4%) of the 251 tumors with negative immunostaining (scored as 0/1+) showed *HER2* amplification. According to the recommended HER2 testing guidelines for gastric cancer, 36 (11.8%, 18 IHC 3+ and 18 IHC 2+, and *HER2* amplified) cases were interpreted as HER2-positive and 270 (88.2%) cases were interpreted as HER2-negative.

### Clinicopathological Characteristics of the Cohort

The clinicopathological characteristics of the 306 GBC cases are displayed in Table 2. The male/female ratio was 1:1.49 (123/183), and the median age was 65 years (range = 27–91 years). Of 306 GBC cases, 223 (72.9%) were AC; 27 (8.8%) were squamous cell carcinoma (SCC)/adenosquamous carcinoma (ASC); 20 (6.5%) were intracystic papillary neoplasm with high grade (ICPN-HG), biliary intraepithelial neoplasia of high grade (BilIN-HG), or tumor *in situ* (Tis); 16 (5.2%) were neuroendocrine neoplasm (NEN)/mixed neuroendocrine/non-neuroendocrine neoplasm (MiNEN); 10 (3.3%) were poor cohesive carcinoma/signet ring cell carcinoma (por/sig); 6 (2.0%) were undifferentiated carcinoma; and 4 (1.3%) were MAC. Among 223 ACs, 62 (27.8%) were papillary carcinomas or carcinomas with partial papillary structures. Regarding the 8th edition of the AJCC cancer staging scheme, 32 (10.5%) cases were in stages 0–I, 71 (23.2%) in stage II, 122 (39.9%) in stage III, and 81 (26.5%) were in stage IV. Two hundred and fifteen patients (70.3%) underwent radical surgery, while 91 (29.7%) had non-radical surgery or biopsy. Fifteen patients had competing malignancies either simultaneously or heterochronously.

**TABLE 2:** Comparison of the clinicopathological features between a HER2-positive and a HER2-negative status

Characteristics, *n* (%)	Total (*n* = 306)	HER2 status		
		Negative(*n* = 270)	Positive(*n* = 36)	*p*-value
Median age (years)	65	64.5	65.5	0.652
Sex
Male	123 (40.2)	108 (40.0)	15 (41.7)	0.848
Female	183 (59.8)	162 (60.0)	21 (58.3)
Histopathological classification
AC	223 (72.9)	187 (69.3)	36 (100)	0.034
ASC/SCC	27 (8.8)	27 (10.0)	0	
Hg/Tis	20 (6.5)	20 (7.4)	0	
MiNEN/NEN	16 (5.2)	16 (5.9)	0	
por/sig	10 (3.3)	10 (3.7)	0	
Undifferentiated CA	6 (2.0)	6 (2.2)	0	
MAC	4 (1.3)	4 (1.5)	0	
Clinical stage
0–I	32 (10.5)	30 (11.1)	2 (5.6)	0.696
IIA–IIB	71 (23.2)	60 (22.2)	11 (30.6)	
IIIA–IIIB	122 (39.9)	107 (39.7)	15 (41.7)	
IVA–IVB	81 (26.5)	73 (27.0)	8 (22.2)	
Type of surgery
Curative	215 (70.3)	188 (69.6)	27 (75.0)	0.508
Palliative/biopsy	1 (29.7)	82 (30.4)	9 (25.0)

*HER2*, human epidermal growth factor receptor 2; *AC*, adenocarcinoma; *ASC*, adenosquamous carcinoma; *SCC*, squamous cell carcinoma; *Hg*, intraepithelial neoplasia of high grade; *Tis*, tumor *in situ*. *MiNEN*, mixed neuroendocrine/non-neuroendocrine neoplasm; *NEN*, neuroendocrine neoplasm; *por/sig*, poor cohesive carcinoma/signet ring cell carcinoma; *MAC*, mucinous adenocarcinoma. N represents the number of samples; P<0.05 indicates the statistical significance.

### Correlation Between HER2 and Clinicopathological Features

The clinicopathological differences between GBC with or without a HER2-positive status are displayed in Table 2. The ratio of HER2-positive status in AC (not including HG/Tis, por/sig, or MAC) was 16.1% (36/223). All GBC with *HER2* amplification was AC (36/36, 100%). SCC/ASC, por/sig, MAC, undifferentiated carcinoma, and NEN/MiNEN were HER2-negative. No statistical differences were observed between the HER2-positive and HER2-negative cases with respect to age, sex, surgery type, or clinical stage.

**FIGURE 1:**
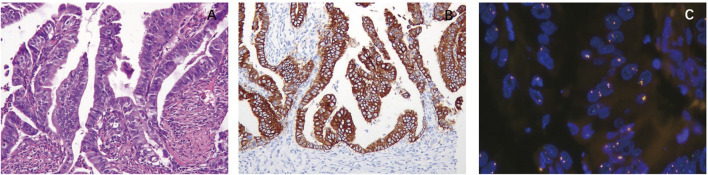
Correlation between morphology and HER2 expression and amplification. **(A)** Papillary adenocarcinoma (HE, ×20). The presence of papillary structure in adenocarcinoma (AC) was associated with HER2 positivity. **(B)** Papillary adenocarcinoma. HER2 immunohistochemistry (IHC) score of 3+ shows strong complete membranous reactivity in all tumor cells. **(C)** Papillary adenocarcinoma. Tumor cells exhibited *HER2* amplification.

The presence of papillary structure in AC was associated with HER2 positivity. Among 223 ACs, 62 cases showed papillary structure or partial papillary structure with a HER2-positive ratio of 41.9% (26/62) (Figures 1A–C), while 161 cases showed no papillary structure with a HER2-positive ratio of 6.2% (10/161, *p* = 0.001).

### Survival Analysis

**FIGURE 2:**
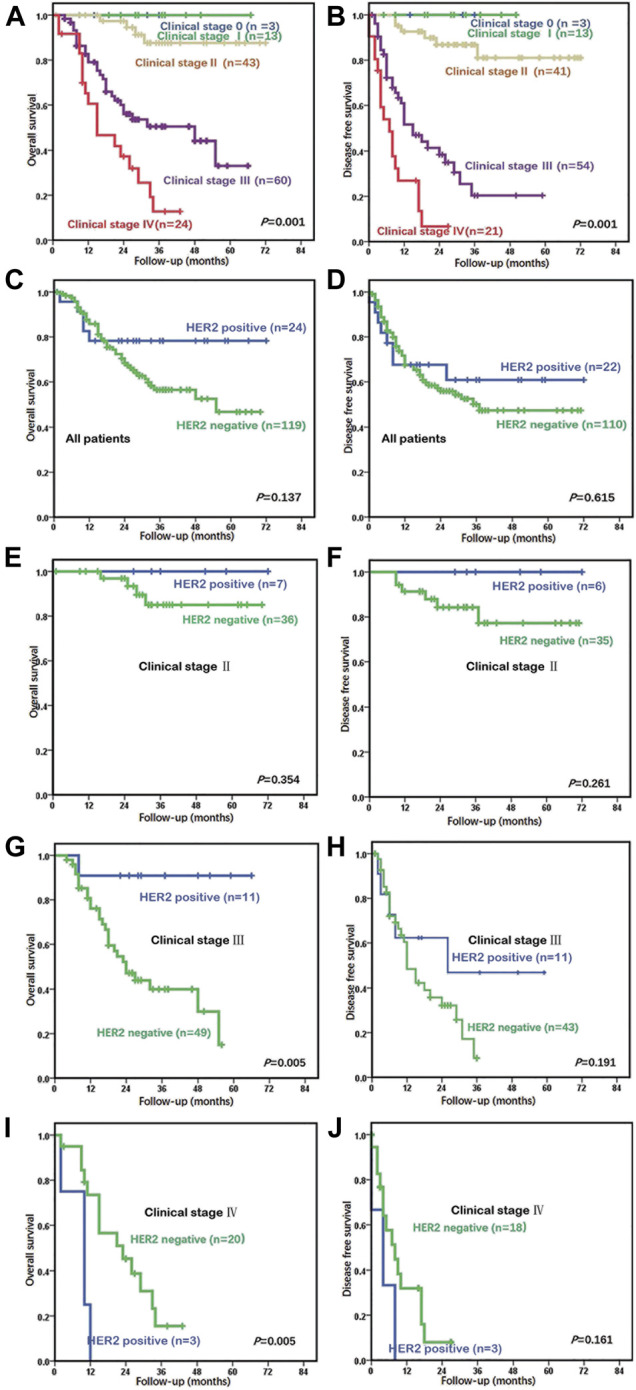
Relationship between prognosis and clinical stages and HER2 status in adenocarcinoma (AC) patients who underwent curative surgeries. **(A)** Clinical stage was markedly related to overall survival (OS) (*p* = 0.001) in all 143 patients. The median OS was 48 months in stage III and was 15 months in stage IV. **(B)** Clinical stage was related to disease-free survival (DFS) (*p* = 0.001). The median DFS was 15 months in stage III and was 7 months in stage IV. **(C)** In the univariate analysis of all 143 patients, those with HER2 positivity tended to have better OS compared to those with a HER2-negative status, but the finding was not significant (*p* = 0.137). **(D)** In the univariate analysis of 143 patients, those with HER2 positivity did not show worse or better DFS in comparison to patients with a HER2-negative status (*p* = 0.615). **(E)** In stage II, the prognostic outcome of the HER2-positive group was similar to that of the HER2-negative group (*p* = 0.354). **(F)** In stage II, the DFS of the HER2-positive group was also similar to that of the HER2-negative group (*p* = 0.261). **(G)** The HER2-positive group had low mortality in stage III (*p* = 0.005). The median OS was 34 months in the HER2-negative group, but this has not been reached by the HER2-positive group. **(H)** The median DFS was 12 months in the HER2-negative group and was 26 months in the HER2-positive group (*p* = 0.191). **(I)** The HER2-positive group had higher mortality at stage IV (*p* = 0.005) compared to the HER2-negative group (median OS of 10 and 23 months, respectively). **(J)** The HER2-positive group had shorter DFS in stage IV (*p* = 0.161) compared to the HER2-negative group (median DFS of 4 and 8 months, respectively).

As most HER2-positive cases were AC (not including por/sig or MAC), only primary AC cases who underwent radical surgery without neoadjuvant radiation and/or chemotherapy and did not have competing malignancies were included in the survival analysis. A total of 146 cases of primary AC after radical resection were included in the survival analysis. The survival data of 143 cases were available, including 119 HER2-negative and 24 HER2-positive patients. The next period was 1–72 months. The median survival time was not reached, and the 5-year survival rate was 52.9%. Also, clinical stage was significantly correlated with OS and disease-free survival (DFS) (*p* = 0.001; Figures 2A, B). The median survival time and DFS were 48 and 15 months in stage III and were 15 and 7 months in stage IV, respectively.

Patients with HER2 positivity did not have poorer or better OS or DFS than patients with a HER2-negative status, according to the univariate analysis (*p* = 0.137, *p* = 0.615) (Figures 2C, D). The HER2-positive group showed a trend toward higher mortality before 20 months and lower mortality after 20 months than did the HER2-negative group. The prognosis of the HER2-positive and HER2-negative groups in stages 0–I, II, III, and IV were compared. All patients in stages 0–I survived, regardless of whether HER2 was positive or not. In stage II, the prognostic outcome of the HER2-positive group was similar to that of the HER2-negative group (*p* = 0.354, *p* = 0.261) (Figures 2E, F). Compared with the HER2-negative group, the HER2-positive group had longer OS and DFS in stage III (*p* = 0.005, *p* = 0.191) (Figures 2G, H) and shorter OS and DFS in stage IV (*p* = 0.005, *p* = 0.161) (Figures 2I, J).

## Discussion

GBC is the most prevalent biliary tract cancer and is the fifth most frequent digestive tract cancer globally (Misra et al., 2003). As GBC patients at an early stage do not have obvious symptoms or signs, most are diagnosed at a late stage. GBC is not sensitive to chemotherapy or radiotherapy, and the prognosis of patients at an advanced stage is poor. It is known that HER2 is a prognostic and therapeutic target in gastric, breast, and colon cancer (Meric-Bernstam et al., 2019). HER2 status was evaluated in GBC and its prognostic value assessed.

The HER2 status in GBC showed considerable heterogeneity, and the frequency of HER2 positivity varied from 0% to 25% in different studies (Albrecht et al., 2020; Matsuyama et al., 2004; Nakazawa et al., 2005; Chaube et al., 2006; Kawamoto et al., 2007; Puhalla et al., 2007; Harder et al., 2009; Shafizadeh et al., 2010; Kumari et al., 2012; Toledo et al., 2012; Roa et al., 2014; Moy et al., 2015; Yoshida et al., 2016). Studies were conducted with IHC or IHC combined with ISH with different cutoff values from 2004 to 2019 on GBC samples of different sizes (6–221). The differences in the positive ratios may have been caused by variations in the detection methods used, cutoff values, sample sizes, and geographic and ethnic differences. Herein, HER2 status was evaluated in a large Chinese cohort based on the recommended testing guidelines for gastric cancer using a combination of IHC and FISH. This study avoided the problems common before 2011, such as the lack of approved antibodies or the inconsistent interpretation standards. The HER2-positive ratios were 11.8% (in all GBCs) and 16.1% (in ACs), which are similar to the results of studies with a larger sample size from Japan (16.6%, *n* = 221, in 2016) (Yoshida et al., 2016) and are slightly higher than that of India (9.6%, *n* = 104, in 2012) (Kumari et al., 2012) and Chile (12.8%, *n* = 187, in 2014) (Roa et al., 2014), but differed from that of Germany (5.4%, *n* = 186, in 2019) (Albrecht et al., 2020). We speculated that the differences may have been caused by race and/or geography. In this research, TMAs were used as the research object of HER2 expression in GBC. Although TMAs can assess the status of HER2 in GBC, there is still a certain possibility of false positives or negatives. A previous study assessed the sampling errors in specimens of biopsy size in gastric cancer by contrasting tissue sections and the corresponding TMAs (Warneke et al., 2013) and found a false-negative rate of 24% and a false-positive rate of 3% for TMAs. This indicates that the HER2-positive ratio may be higher than 16.1% in patients with AC.

Papillary structure in carcinoma is markedly associated with HER2 positivity. In this study, the HER2-positive ratio was 41.9% in papillary AC or AC with papillary structure and 6.2% in common ACs. Gastric cancer (Oono et al., 2018) and lung adenocarcinoma (Kim et al., 2017) have also reported the same phenomenon. The HER2-positive rate of papillary adenocarcinomas was especially high in gastric cancer (62%, 8/13, *p* = 0.023) (Oono et al., 2018) and lung cancer (17%, 7/41, *p* = 0.029) (Kim et al., 2017). Several studies have also evaluated the relationship between HER2 and papillary structures in GBC (Yoshida et al., 2016; Albrecht et al., 2020). Due to the small size of papillary AC (*n* = 10) or because these studies evaluated mixed tumors with other types of cancer, no statistical relationship was found. In this study, 62 (27.8%) were papillary carcinomas or carcinomas with partial papillary structures among 223 ACs. The HER2-positive ratio of 41.9% (26/62) was significantly higher than that of ACs without papillary structures. This indicates that a HER2 test should be applied in gallbladder ACs with papillary structures to investigate targeted therapies.

In most cases, *HER2* amplification and protein overexpression have been consistent; however, abnormalities have been observed, wherein the overexpression of HER2 protein was inconsistent with the expression of the gene. In this study, 6 (2.4%) of the 251 tumors with negative immunostaining (scored as 0/1+) showed *HER2* amplification. In other words, in some cases, *HER2* amplification may not lead to protein overexpression, which was also observed in gastric (Hofmann et al., 2008) and breast cancer (Todorovic-Rakovic et al., 2005). Similarly, a considerable proportion of human cancers with moderate overexpression of HER2 does not show gene amplification (Jimenez et al., 2000; Bofin et al., 2004). These findings indicate that other mechanisms are involved in the regulation of HER2 expression. Zuo et al. reported that forkhead box P3 (*FOXP3*) gene alterations (deletion, somatic mutations of functional significance, and downregulation) are common in breast cancer samples, and they found that these alterations correlated significantly with HER2 overexpression regardless of the status of *HER2* amplification (Zuo et al., 2007). These data demonstrate that *FOXP3* is an important oncogene that may regulate the *HER2* gene (Zuo et al., 2007).

Although HER2 is a separate prognostic factor in breast (Loibl and Gianni, 2017) and gastric cancer (Gravalos and Jimeno, 2008), a HER2-positive status does not show prognostic value for the OS or DFS of GBC. However, some studies found that the HER2-positive group tended to have shorter OS and/or DFS (Albrecht et al., 2020; Roa et al., 2014; Yoshida et al., 2016). The prognosis of the same tumor at different stages was completely different, so the clinical stage is also a separate prognostic factor. In this study, no patient in stages 0–I died of GBC, and the median survival time of stage II patients was not reached, while those of stage III and IV patients were 48 and 15 months, respectively. Thus, tumors at different stages may be regarded as different subgroups; they have different molecular events. Similarly, a molecule may have different roles at different stages of the tumor (Xu et al., 2017a). Here, the prognostic value of HER2 was analyzed for GBC at different stages. HER2 positivity was not prognostic for OS or DFS in stages 0–II, with low mortality in stage III (*p* = 0.005) and high mortality in stage IV (*p* = 0.005). These results suggest that HER2 might have different roles alone or in combination with other molecules at different stages during the development of GBC. The same phenomenon has been observed in the prognostic significance of induced myeloid leukemia cell differentiation one copy number gain in esophageal squamous cell carcinoma (Xu et al., 2017b). Whether this finding is constant or accidental requires further research.

The limitation of this study is that it cannot reflect the actual status of HER2 heterogeneity in GBC. As TMA was used in this study, the samples we chose did not fully reflect the characteristics of the tumors, and heterogeneity is an important feature of tumors. Another limitation is that this is a retrospective study. Since none of the patients included in this study received anti-HER2 therapy, its impact on patients cannot be assessed. The study was unable to evaluate the effects of anti-HER2 therapies on these patients. However, HER2 status is the basis of anti-HER2 therapies in GBC, and this study provides evidence for treatment.

In conclusion, the frequency of HER2 positivity was reported in a large, well-characterized Chinese cohort of patients with GBC with the recommended testing guidelines for gastric cancer using a combination of IHC and FISH. The HER2-positive rate was 16.1% in AC, and more than 40% in papillary AC or AC with papillary structure. HER2 positivity had a bidirectional prognostic significance in patients with GBC at different clinical stages. The above results provide the basis for targeted treatment for HER2.

## Data Availability

The original contributions presented in the study are included in the article/Supplementary Material. Further inquiries can be directed to the corresponding author.
